# Calibration of variant effect predictors for in-frame indels for clinical variant classification

**DOI:** 10.1093/bib/bbaf631.073

**Published:** 2025-12-12

**Authors:** Haneen Abderrazzaq, Mugdha Singh, Timothy Bergquist, Vikas Pejaver, Anne O’Donnell-Luria, Predrag Radivojac

**Affiliations:** Northeastern University; Broad Institute of MIT and Harvard, Boston Children’s Hospital; Icahn School of Medicine at Mount Sinai; Icahn School of Medicine at Mount Sinai; Broad Institute of MIT and Harvard, Boston Children’s Hospital; Northeastern University

## Abstract

Insertions and deletions (indels) are the second most common type of variant in humans and are associated with a wide range of functional consequences. Despite their biological importance, indels, particularly non-frameshifting ones, remain understudied. While many computational predictors for missense variants have been rigorously evaluated, the clinical utility of tools for non-frameshifting indels remains uncertain. In this work, we calibrate non- frameshifting indel predictors for clinical variant classification, using a previously established framework developed for calibrating missense predictors. We constructed a dataset of high- confidence in-frame indel variants from ClinVar and gnomAD and estimated the prior probability of pathogenicity for all in-frame indels, as well as for insertions and deletions separately. Using a statistical framework based on local posterior probabilities, we then established score thresholds for several computational tools, including MutPred-Indel, VEST-indel, and CADD, corresponding to different levels of evidence for pathogenicity and benignity according to ACMG/AMP guidelines. These guidelines outline criteria for the clinical classification of variants, where different types of evidence (functional, population, computational, etc.) are weighted by strength (supporting, moderate, strong, etc.) and assigned point values that are summed to determine whether a variant is pathogenic, benign, or of uncertain significance. Distinct from several missense predictors which can reach the strong (+4 points) level of evidence, we find that most in-frame indel predictors reach the moderate (+2 points) or supporting (+1 point) evidence levels, demonstrating their clinical value, while highlighting the need for improved tools for indel interpretation.

